# Significant co-expression of putative cancer stem cell markers, EpCAM and CD166, correlates with tumor stage and invasive behavior in colorectal cancer

**DOI:** 10.1186/s12957-021-02469-y

**Published:** 2022-01-11

**Authors:** Elham Kalantari, Tahereh Taheri, Saba Fata, Maryam Abolhasani, Mitra Mehrazma, Zahra Madjd, Mojgan Asgari

**Affiliations:** 1 Oncopathology Research Center, Iran University of Medical Sciences, Hemmat Street (Highway), Next to Milad Tower, Tehran, 14496-14530 Iran; 2 Department of Pathology, Iran University of Medical Sciences, Tehran, Iran; 3 Department of Pathology, Hasheminejad kidney Center, Iran University of Medical Sciences, Tehran, Iran; 4 Department of Molecular Medicine, Faculty of Advanced Technologies in Medicine, Iran University of Medical Sciences, Tehran, Iran

**Keywords:** Cancer stem cells, EpCAM, CD166, Colorectal cancer, Tissue microarray

## Abstract

**Background:**

The crucial oncogenic role of cancer stem cells (CSCs) in tumor maintenance, progression, drug resistance, and relapse has been clarified in different cancers, particularly in colorectal cancer (CRC). The current study was conducted to evaluate the co-expression pattern and clinical significance of epithelial cell adhesion molecules (EpCAM) and activated leukocyte cell adhesion (CD166 or ALCAM) in CRC patients.

**Methods:**

This study was carried out on 458 paraffin-embedded CRC specimens by immunohistochemistry on tissue microarray (TMA) slides.

**Results:**

Elevated expression of EpCAM and CD166 was observed in 61.5% (246/427) and 40.5% (164/405) of CRC cases. Our analysis showed a significant positive association of EpCAM expression with tumor size (*P* = 0.02), tumor stage (*P* = 0.007), tumor differentiate (*P* = 0.005), vascular (*P* = 0.01), neural (*P* = 0.01), and lymph node (*P* = 0.001) invasion. There were no significant differences between CD166 expression and clinicopathological parameters. Moreover, the combined analysis demonstrated a reciprocal significant correlation between EpCAM and CD166 expression (*P* = 0.02). Interestingly, there was a significant positive correlation between EpCAM/CD166 phenotypes expression and tumor stage (*P* = 0.03), tumor differentiation (*P* = 0.05), neural, and lymph node invasion (*P* =0.01).

**Conclusions:**

The significant correlation of EpCAM and CD166 expression and their association with tumor progression and aggressive behavior is the reason for the suggestion of these two CSC markers as promising targets to promote novel effective targeted-therapy strategies for cancer treatment in the present study.

**Supplementary Information:**

The online version contains supplementary material available at 10.1186/s12957-021-02469-y.

## Background

Colorectal cancer (CRC) is the fourth most common cancer and a leading cause of death, worldwide [1]. CRC is caused by a complicated multistep molecular etiology including various genetic and epigenetic alterations [2]. With respect to the tumor stage, more than 50% of patients are diagnosed with stage III disease, while only 25% showed stages I and II [3, 4]. Therefore, recurrence and distant metastasis are the main findings in patients with higher stages [5]. Surgical resection is the most common and first treatments in CRC cases besides chemotherapy and radiotherapy. In this regard, identification and characterization of prognostic cancer biomarkers can pave the way to early treatment and inhibition of tumor progression by targeted-therapy strategies [6, 7]. Increasing evidence has highlighted the role of cancer stem cells (CSCs) in tumor initiation, development, recurrence, metastasis, and drug resistance that are identified by their surface markers. The wide range of CSC markers is recognized in different solid and hematopoietic tumors [8–15]. Epithelial cell adhesion molecules (EPCAM or EpCAM) and CD166, leukocyte cell adhesion molecule (ALCAM), are two transmembrane glycoproteins which are involved in adhesion interactions between cells, while expressed in malignant cells [16, 17].

The biological role of EpCAM has been proved in most solid tumors, including colorectal cancer [18–20]. Because of the controversial activity of this marker, different expression patterns and correlation with survival have been reported [21]. EpCAM plays a different role as an oncogenic and/or tumor suppressor gene depending on its microenvironment in different tumor types. Its role as a homophilic intercellular adhesion molecule has been reported and justifies its anti-metastatic function and down regulation of EpCAM in metastases of renal clear cell carcinomas and thyroid carcinoma [22, 23]. The abovementioned points are evidence that show the significant correlation of lower expression of EpCAM with improved patients’ survival [24–26]. In contrast, based on EpCAM activity on cell signaling pathways, its invasive functions in tumor growth and progression have been suggested in the bladder, gallbladder, breast, prostate, lung, pancreas, and renal cell carcinoma [27–36]. Controversial results have been identified in gastric [37, 38] and colorectal cancer [34, 39].

CD166 protein is a type-1 glycoprotein from the immunoglobulin superfamily, which is known as both putative mesenchymal stem cell marker and maintenance of CSCs characteristic including tumor initiation, proliferation, and invasion has been reported in different cancers such as breast, ovarian, prostate, and CRC [40–43]. Moreover, the correlation of overexpression of this marker with survival and tumor regression highlighted the CD166 as a potential prognostic marker in esophageal squamous cell carcinoma and CRC patients [44–46]. However, there have been some controversial results considering the correlation of CD166 expression with clinical significance in CRC specimens [43, 47, 48].

Regarding the above description and contradictory findings of EpCAM and CD166 expression in the previous studies, this study was conducted to evaluate the co-expression pattern of EpCAM and CD166 and its association with clinicopathological profile in a large series of CRC patients using tissue microarray (TMA)-based immunohistochemistry (IHC) analysis.

## Methods

### Sample collection

This study comprised 458 archival paraffin-embedded CRC samples and 30 matched adjacent normal tissues collected from Hasheminejad, Rasool Akram, and Firoozgar hospitals between 2009 and 2015 in Tehran, Iran. All histopathological data was recorded from the corresponding hematoxylin and eosin slides including sex, age, tumor size, tumor location, TNM staging classification, tumor differentiation, distance metastases, and the presence of vascular, neural, and lymphnode invasion. None of the CRC patients in this study had received neoadjuvant treatment before surgery.

### Tissue microarray (TMA) construction

Hematoxylin and eosin (H&E)-stained slides were examined by an expert pathologist to spot the representative area of each tumor tissue, as described previously [8, 36, 49, 50]. In brief, each TMA recipient block contains almost 65 tissue samples with a diameter of 0.6 mm which were constructed in three copies for each specimen; final scoring was evaluated by the mean scoring of three cores. Subsequently, the TMA blocks were cut into 4 m thin serial sections and transferred onto positively charged TMA slides (Superfrost plus, Thermo Scientific, Germany). In TMA-based studies, to overcome the heterogeneity of protein expression, we analyzed three cores of each specimen to elevate the accuracy and validity of the experiment [51].

### Immunohistochemistry (IHC)

Formalin-fixed, paraffin-embedded sections of the TMA-constructed slides were stained using the Biopharmadex kit (Link-Envision; KL5007, Germany), as described previously [52, 53]. After dewaxation at 60 °C for 30 min followed by rehydration steps, the samples were incubated overnight with anti-EpCAM (1:1000 dilution, ab124825; Abcam, UK) and anti-CD166 (1/500, ab109215; Abcam, UK) at 4 °C. Antigen retrieval was done by autoclave for 11 min; anti-EpCAM in citrate buffer (pH = 6.0) and anti-CD166 in Tris EDTA buffer (pH = 8.0). The sections were then treated with the secondary antibody, ^TM^Mouse/Rabbit Poly Vue HRP/DAB detection kit (standard EnVision-HRP kit (Bio pharmadx)), at room temperature (RT). This was followed by visualization with 3; 3′-diaminobenzidine (DAB) substrate as a chromogen for 3 min at RT; the sections were counterstained with hematoxylin for 15 min, were dehydrated, and were finally mounted. Human colon adenocarcinoma and liver tissues were selected as the positive controls for anti-EpCAM and anti-CD166, respectively. Replacement of the primary antibodies by preimmune rabbit IgG and Tris Buffer Saline (TBS, pH: 7.4) wash buffer was used as the negative controls [54, 55].

### Scoring system of TMA slides

A semi-quantitative system was used by two pathologists to score each TMA tissue section with no prior knowledge of clinicopathologic parameters of samples. Immunostaining of EpCAM and CD166 was evaluated as described previously [8]. Each marker expression was scored independently and the final scoring assessment was carried out with reinvestigation of the overall distribution of the tumor cells at 10× magnification. Positive cells were then assessed, semi-quantitatively, at higher magnifications (20× or 40×). The intensity of immunostaining was divided into groups 0, 1, 2, and 3 from negative to strong staining. The percentage of positive cells was valued semi-quantitatively and scored as 0–100%. The histochemical score (*H*-score) was obtained by multiplying the intensity (0–3) and percentage scores (0–100%), and generated scores of 1–100, 100–200, and 200–300 [56]. The mean *H*-score (=196) was chosen as the cutoff point for Anti-EpCAM and (= 83) for anti-CD166. The specimens with *H*-score ≤ 196 and ≤ 83 were considered to be low EpCAM and CD166 expressing tissues, and the specimens with *H*-score > 196 and > 83 were considered to be high EpCAM and CD166 tissues [49, 57].

### Statistical analysis

Statistical analysis was performed using the SPSS software version 22 (SPSS, Chicago, IL, USA). The association between EpCAM and CD166 expression and clinicopathological features was determined by logistic regression, Pearson’s chi-square, and Spearman’s correlation coefficient test. A *P* value < 0.05 was considered statistically significant.

## Results

Clinicopathological characteristics of all cases are summarized in Table 1. Patients had a mean age of 60 ± 14.7 years, and males had higher proportion of the distribution of gender with 51.5% (236/458). Based on the tumor size (mean = 5 cm); 66% of samples had less than 5 cm in size. Of all patients, 63.5% had moderate/poor differentiation and 36.5% had well differentiated. Seventy-one (16%) specimens had stage I, 172 (38%) stage IIA, 21 (5%) stage IIB, 74 (17%) stage IIIA, 68 (15%) stage IIIB, 17 (4%) stage IIIC, and 21 (5%) had stage IVA.

**Table 1:** Statistical association of EpCAM and CD166 expression with clinicopathological parameters in colorectal cancer specimens (Pearson *χ*^2^). The bold values are statistically significant

Variables	Total no. (%)	EpCAM expression (mean H-score = 196)		*P* value	CD166 expression (mean H-score = 83)		*P* value
		Low	High		Low	High	
**Mean age years**							
60 ≥	241 (52.5)	72 (34)	140 (66)	**0.03**	127 (60)	85 (40)	0.47
60 <	218 (47.5)	88 (43)	115 (57)		114 (59)	79 (41)	
**Gender**							
Male	236 (51.5)	87 (40)	130 (60)	0.26	117 (56)	92 (44)	0.08
Female	222 (48.5)	72 (36.5)	125 (63.5)		123 (63)	79 (37)	
**Tumor size (cm)**							
5 ≥	300 (66)	96 (35)	175 (65)	**0.02**	158 (59)	109 (41)	0.5
5 <	154 (34)	64 (46)	76 (54)		78 (58.5)	55 (41.5)	
**TNM stage**							
I	71 (16)	20 (31)	45 (69)	**0.007**	46 (70)	20 (30)	0.2
IIA	172 (38)	48 (30.5)	109 (69.5)		81 (52.5)	73 (47.5)	
IIB	21 (5)	9 (45)	11 (55)		12 (60)	8 (40)	
IIIA	76 (17)	36 (53)	32 (47)		33 (53)	29 (47)	
IIIB	68 (15)	29 (48)	31 (52)		39 (68.5)	18 (31.5)	
IIIC	17 (4)	9 (60)	6 (40)		9 (69)	4 (31)	
IVA	21 (5)	7 (36.5)	12 (63.5)		14 (67)	7 (33)	
**Tumor location**							
Cecum	70 (16.5)	29 (44)	37 (56)	0.09	37 (58)	27 (42)	0.29
Sigmoid	140 (34)	36 (28)	92 (72)		73 (58.5)	52 (41.5)	
Rectom	114 (27)	45 (48.5)	48 (51.5)		64 (69)	29 (31)	
Colon ascending	34 (8)	11 (34.5)	21 (65.5)		20 (67)	10 (33)	
Colon transvers	29 (7)	9 (32)	19 (68)		13 (46.5)	15 (53.5)	
Colon descending	12 (3)	5 (45.5)	6 (54.5)		5 (42)	7 (58)	
Rectosigmoid	18 (4.5)	8 (44.5)	10 (55.5)		11 (65)	6 (35)	
**Tumor differentiation**							
Well	165 (36.5)	45 (30)	103 (70)	**0.005**	93 (63)	55 (37)	0.17
Moderate/poor	289 (63.5)	115 (43.5)	148 (56.5)		145 (57.5)	107 (42.5)	
**Distant metastasis**							
Positive	25 (6)	7 (30)	16 (70)	0.29	17 (68)	8 (32)	0.24
Negative	416 (94)	146 (38.5)	232 (61.5)		215 (59)	150 (41)	
**Neural invasion**							
Positive	90 (20)	41 (52.5)	38 (47.5)	**0.01**	47 (69)	22 (31)	0.15
Negative	355 (80)	113 (35)	212 (65)		188 (58)	138 (42)	
**Vascular invasion**							
Positive	69 (15.5)	30 (52.5)	27 (47.5)	**0.01**	37 (65)	20 (35)	0.25
Negative	379 (84.5)	124 (35.5)	224 (64.5)		201 (59)	139 (41)	
**Lymph node invasion**							
Positive	171 (37.5)	74 (48.5)	78 (51.5)	**0.001**	89 (61.5)	56 (38.5)	0.31
Negative	286 (62.5)	84 (32)	177 (68)		151 (58)	108 (42)	

### Expression of EpCAM in colorectal cancer and adjacent normal tissues

**Fig. 1:**
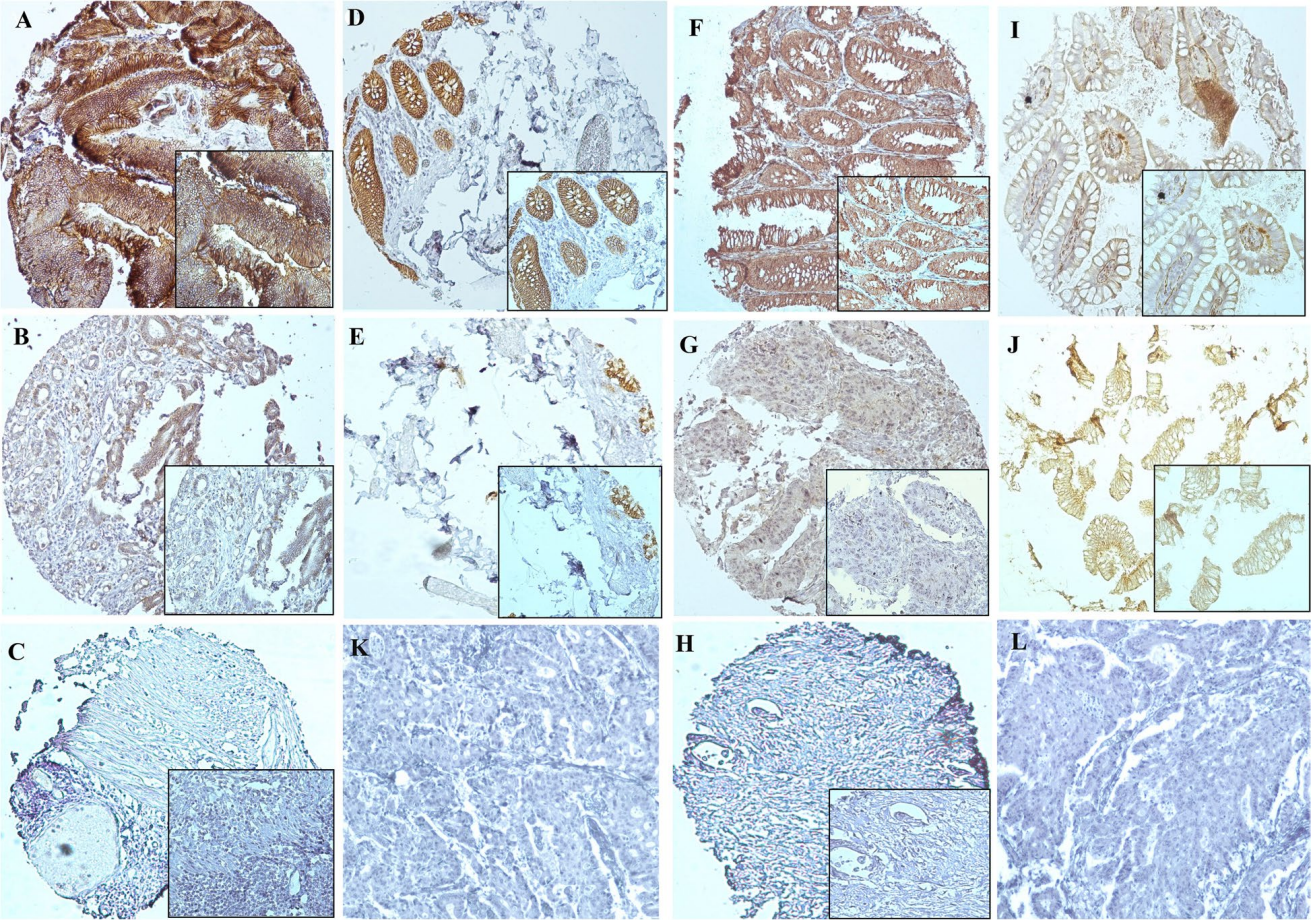
Immunohistochemical staining of EpCAM and CD166 in colorectal cancer specimens and adjacent normal tissues. **A** Higher, **B** lower, and **C** negative expression of EpCAM in colorectal cancer tissue. **D** Higher and **E** lower expression of EpCAM in adjacent normal tissue. **F** Higher, **G** lower, and **H** negative expression of CD166 in colorectal cancer tissues. **I** Higher and **J** lower expression of CD166 in adjacent normal tissue. **K** and **L** preimmune rabbit IgG as negative controls for EpCAM and CD166, respectively (all images were taken at 100× and 200× magnification)

The higher expression of EpCAM observed in CRC samples compared to adjacent normal tissues. Because of technical problems, from all 458 specimens, 415 samples remained for statistics analysis of EpCAM expression. In terms of intensity, membranous expression of EpCAM showed strong (+3) in 150 (36%), moderate (+2) in 170 (41%), weak (+1) in 89 (21.5%), and negative (0) in 6 (1.5%) specimens. Based on *H*-score scoring, 255 (61.5%) of all the samples had higher and 160 (38.5%) had lower expression of EpCAM. From 30 adjacent normal tissues, 6.5%, 16.5%, and 77% of samples demonstrated strong, moderate, and weak intensity staining of EpCAM expression, respectively. Moreover, in terms of *H*-score; only one sample represented the elevated expression of EpCAM and 29 (96.5%) of normal specimens displayed lower immunoreactivity of EpCAM (Fig. 1, Table 2).

**Table 2:** Expression of EpCAM and CD166 (intensity and H-score) in colorectal cancer and adjacent normal tissues

Scoring system	Carcinoma		Normal	
	EpCAM	CD166	EpCAM	CD166
	*N* (%)	*N* (%)	*N* (%)	*N* (%)
**Intensity of staining**				
Strong (+3)	150 (36)	25 (6)	2 (6.5)	1 (3.5)
Moderate (+2)	170 (41)	112 (27)	5 (16.5)	7 (23.5)
Weak (+1)	89 (21.5)	185 (46)	23 (77)	16 (53)
Negative (0)	6 (1.5)	83 (21)	0 (0)	6 (20)
**H-score**				
High	255 (61.5)	164 (40.5)	1 (3.5)	8 (27)
Low	160 (38.5)	241 (59.5)	29 (96.5)	22 (73)
Total	415	405	30	30

### Clinicopathological significance of EpCAM expression

**Fig. 2:**
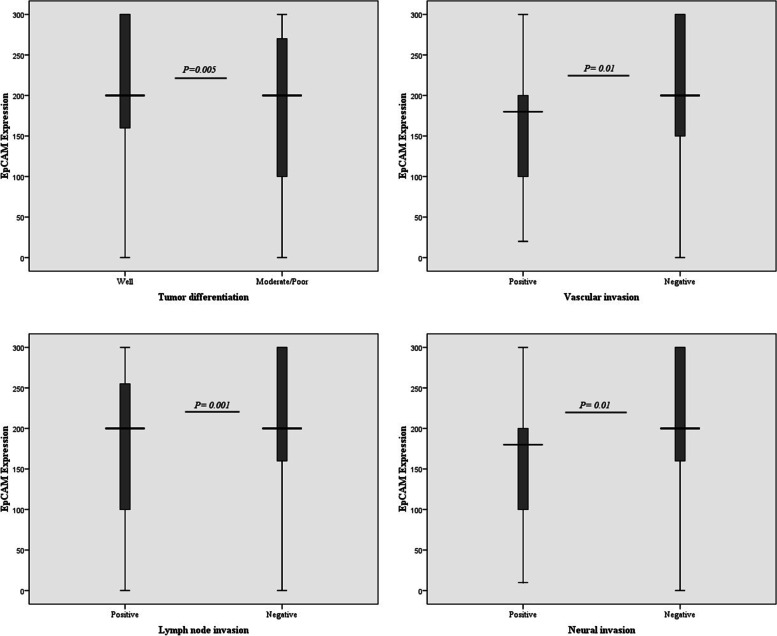
Box-plot diagram of EpCAM and CD166 expressions in tumor differentiation, vascular, neural, and lymph node involvement in colorectal cancer specimens. Based on the standard definitions, each box-plot shows the median (bold line), interquartile lines (box), and outlier observation (circle)

Univariate analysis showed a positive significant association between tumor size, tumor stage, tumor differentiation, vascular, neural, and lymph node invasion and higher expression of EpCAM. Moreover, logistic analysis demonstrated a positive significant correlation between age, tumor stage, and tumor differentiation and higher expression of EpCAM (Supplementary Table 1). The overexpression of EpCAM was demonstrated in 54% of specimens with more than 5 cm tumor size (*P* = 0.02). In terms of tumor stage, 45 (69%), 109 (69.5%), 11 (55%), 32 (47%), 31 (52%), 6 (40%), and 12 (63.5%) of stage I, IIA, IIB, IIIA, IIIB, IIIC, and IVA displayed higher expression of EpCAM, respectively (*P* = 0.007). Out of 263 moderate/poor differentiated samples 148 (56.5%) and from 148 well differentiated cases, 103 (70%) displayed higher expression of EpCAM (*P* = 0.005). Of 57 samples with positive vascular invasion, 27 (47.5%) had higher expression of EpCAM (*P* = 0.01). From 79 positive neural invasion patients 38 (47.5%, *P* = 0.01), and from 152 positive lymph node invasion, 78 (51.5%) of samples showed higher level of EpCAM expression (*P* = 0.001); Fig. 2 and Table 1 displayed the correlation of EpCAM expression with all clinicopathological features.

### Expression of CD166 in colorectal cancer and adjacent normal tissues

There was no significant difference in CD166 expression pattern between CRC samples and adjacent normal tissues. Upon IHC staining, CD166 expression mainly localized in membrane and partially in cytoplasmic area of tumor cells. In terms of intensity, from 405 specimens, only 25 (6%) showed strong intensity of staining; moderate, weak, and negative expression of CD166 was found in 112 (27%), 185 (46%), and 83 (21%), respectively. Regarding *H*-score scoring system, a higher immunoreactivity of CD166 was seen in 164 (40.5%) of samples and a lower CD166 expression was observed in 241 (59.5%) of specimens. Scoring of 30 adjacent normal tissues demonstrated strong, moderate, weak, and negative intensity staining of CD166 expression in 1 (3.5%), 7 (23.5%), 16 (53%), and 6 (20) specimens, respectively. Furthermore, the higher immunoreactivity of CD66 expression displayed in 8 (27%) normal sample and 22 (73%) showed lower expression of CD166 (Fig. 1, Table 2). Statistics analysis showed that there were no significant association between CD166 expression and clinicopathological features of samples. All data was collected and summarized in Table 1 and Supplementary Table 1.

### Combined analysis of EpCAM/CD166 expression

Immunohistochemically expression pattern of both EpCAM and CD166 markers suggested a reciprocal significant correlation between two markers (*P* = 0.02). Among 360 combined cases, 77 (21.5%) specimens had EpCAM^low^/CD166^low^ phenotype, 127 (35.5%) samples showed EpCAM^high^/CD166^low^ phenotype, 58 (16%) cases had EpCAM^low^/CD166^high^ phenotype, and 98 (27%) samples had EpCAM^high^/CD166^high^ phenotype. The association of EpCAM/CD166 phenotypes expression with clinicopathological characteristics of CRC specimens was examined by one-way ANOVA and Tukey’s post hoc analysis tests. The findings observed a significant direct correlation between EpCAM/CD166 phenotypes expression and tumor stage (*P* = 0.03), tumor differentiation (*P* = 0.05), neural, and lymph node invasion (*P* = 0.01). There were no significant correlation between other EpCAM/CD166 phenotypes and clinicopathological variables (Table 3).

**Table 3:** Statistical association of EpCAM/CD166 phenotypes expression with clinicopathological parameters in colorectal cancer specimens (Pearson *χ*^2^). The bold values are statistically significant

Variables	Total no. (%)	EpCAM/CD166 phenotype expression, no. (%)				*P* value
		EpCAMLow/CD166Low	EpCAMHigh/CD166Low	EpCAMLow/CD166High	EpCAMHigh/CD166High	
**Mean age years**						
60 ≥	184 (51)	32 (17.5)	71 (38.5)	29 (16)	52 (28)	0.2
60 <	176 (49)	45 (25.5)	56 (32)	29 (16.5)	46 (26)	
**Gender**						
Male	192 (53.5)	40 (21)	64 (33.5)	35 (18)	53 (27.5)	0.65
Female	167 (46.5)	36 (21.5)	63 (37.5)	23 (14)	45 (27)	
**Tumor size (cm)**						
5 ≥	239 (67)	47 (20)	90 (37.5)	36 (15)	66 (27.5)	0.26
5 <	117 (33)	30 (25.5)	33 (28)	22 (19)	32 (27.5)	
**TNM stage**						
I	59 (16.5)	12 (20)	28 (47.5)	5 (8.5)	14 (24)	**0.03**
IIA	139 (39)	28 (20)	42 (30)	16 (11.5)	53 (38.5)	
IIB	19 (6)	6 (31.5)	5 (26)	3 (16)	5 (26)	
IIIA	54 (15)	8 (15)	18 (33)	18 (33)	10 (19)	
IIIB	49 (14)	13 (26.5)	20 (41)	11 (22.5)	5 (10)	
IIIC	11 (3)	5 (45.5)	2 (18)	1 (9)	3 (27.5)	
IVA	19 (5.5)	5 (26)	7 (37.5)	2 (10.5)	5 (26)	
**Tumor location**						
Cecum	60 (19)	18 (30)	16 (27)	9 (15)	17 (28)	**0.05**
Sigmoid	113 (34)	16 (14)	46 (41)	14 (12.5)	37 (32.5)	
Rectom	73 (22)	20 (27.5)	27 (37)	17 (23.5)	9 (12)	
Colon ascending	28 (8.5)	8 (29)	11 (39)	1 (3)	8 (29)	
Colon transvers	27 (8)	2 (7.5)	10 (37)	7 (26)	8 (29.5)	
Colon descending	11 (3.5)	2 (19)	3 (27)	3 (27)	3 (27)	
Rectosigmoid	17 (5)	6 (35)	5 (29.5)	1 (6)	5 (29.5)	
**Tumor differentiation**						
Well	132 (37)	25 (19)	54 (41)	13 (10)	40 (30)	**0.05**
Moderate/poor	225 (63)	52 (23)	72 (32)	45 (20)	56 (25)	
**Distant metastasis**						
Positive	23 (6.5)	4 (17.5)	11 (48)	3 (13)	5 (21.5)	0.65
Negative	326 (93.5)	70 (21.5)	113 (35)	54 (16.5)	89 (27)	
**Neural invasion**						
Positive	57 (16.5)	22 (39)	14 (25)	10 (17)	11 (19)	**0.01**
Negative	294 (83.5)	53 (18)	110 (37.5)	47 (16)	84 (28.5)	
**Vascular invasion**						
Positive	45 (13)	12 (27)	14 (31)	11 (24.5)	8 (17.5)	0.18
Negative	308 (87)	63 (20.5)	112 (36.5)	46 (15)	87 (28)	
**Lymph node invasion**						
Positive	126 (35)	29 (23)	44 (35)	29 (23)	24 (19)	**0.01**
Negative	233 (65)	47 (20)	83 (35.5)	29 (12.5)	74 (32)	

## Discussion

Pioneer studies had highlighted the potential function of CSC markers in tumor aggressiveness, drug resistance, and consequently treatment failure in CRC patients after postoperative chemotherapy and/or radiotherapy [53, 58]. Evidence suggests that information regarding EpCAM and CD166 expression and clinical significance are not consistent in different solid tumors [21, 59]. From this point of view, we aimed at evaluating co-expression and the clinical significance of the two putative CR-CSC markers EpCAM and CD166, in a large series of CRC specimens. Our findings showed the higher expression of EpCAM in 61.5% of CRC patients and the direct significant association of EpCAM expression with tumor size (*P* = 0.02), tumor stage (*P* = 0.007), tumor differentiation (*P* = 0.005), and vascular (*P* = 0.01), neural (*P* = 0.01), and lymph node (*P* = 0.001) invasion. Diversity of EpCAM function can cause controversies in expression pattern of this marker in different tumors, especially in CRC cases. Our results are in line with several in vivo and in vitro reports, which have revealed the key role of EpCAM in self-renewal, differentiation, migration, and invasion in different solid tumors [60–62]. Our recent study on clear cell renal cell carcinoma (ccRCC) indicated the higher membranous expression of EpCAM and its direct significant association with nucleolar grade and tumor necrosis. We also found EpCAM to be an independent favorable prognostic marker affecting progression-free survival (PFS) in ccRCC [36]. Previously, Liu et al. represented the tumor progression, aggressiveness, and chemotherapy resistance in CRC tissues with EpCAM+/CD44+ phenotype [63]. The immunohistochemical observation in TMA tissues of Went et al. also demonstrated the significant higher expression of EpCAM protein in high-grade CRC tumors [20]. Zhou and colleagues noted the high expression of EpCAM in colon cancer and its correlation with lower survival rates in 50 tissues by immunohistochemistry [64]. However, there are some other studies in the literature which have suggested the negative association of EpCAM expression with tumor grade, invasion, and lymph node metastasis [48] and noted the correlation of a decreased expression of EpCAM with poor survival and cancer recurrence in CRC patients [21, 39, 65]. The diversity of all of these findings can be due to the different biological functions of EpCAM CSC marker in different tumor types, particularly CRC, as described previously. EpCAM acts as a double-edged sword protein that has oncogenic and tumor suppressive behavior biologically. Cell formation, adhesive structure, and polarity make up the potential traits of EpCAM protein. Although the loss of adhesive structure and cell polarity generally happens in tumor cells, higher expression of this protein has been clarified in tumor cells [21, 60, 66].

In addition, our results revealed that 40.5% of CRC cases had increased levels of CD166 membranous immunoreactivity, and there was no significant correlation with the clinical profile of patients such as clinical stage, distant metastasis, lymph node, neural, and vascular invasion. There is some contentious information regarding the difference between the various localization patterns of CD166 and its relation with demographic features and overall survival of the patient. Because of the different cellular positions of CD166, it is predominantly expressed in cell membrane and partially in cytoplasm [67]. Our findings are consistent with several pieces of evidence suggesting a decreased or no clinical significance of the membranous expression of CD166 in CRC tissues by immunohistochemistry [17, 43, 48]. A comprehensive study of 1420 CRC samples using TMA constructions observed the lower immunoreactivity of CD166 in high grade tumors, larger tumor size, infiltrating tumor border configuration, and overall less survival cases [48]. Tachezy et al. reported the major membranous localization of CD166 in primary tumors versus secondary and distant metastatic tumors and negative significant clinical differences with tumor differentiation grade. They introduced CD166 as a good prognostic marker in CRC patients [43]. A study carried out by Weichert et al. noted the cell membrane expression of CD166 in only 31% of CRC tissues and there were no significant association between CD166 expression and clinicopathological features such as grade, stage, and lymph node invasion; however, their multivariate analysis showed CD166 as an independent poor prognostic marker in CRC specimens [68]. Another study conducted on 110 CRC samples indicated that 64% of primary tumors had positive membranous expressions of CD166, but they found no significant correlation with clinicopathological parameters [47]. In contrast, other researches represented a direct significant correlation of CD166 expression with tumor regression and worse prognosis effects of this marker in preoperative chemoradiotherapy-treated colorectal adenocarcinoma specimens [45]. Evidence confirmed the translocation feature of CD166 from the cell membrane to the cytoplasmic localization by a clathrin-dependent pathway [69]. Interestingly, Amanda et al. evaluated the intracellular and extracellular domain of CD166 by dual stain assay in 105 CRC samples and defined shedding of extracellular expression of CD166 after intracellular localization of this protein. They clarified the correlation of cytoplasmic expression pattern of CD166 with poor prognosis suggesting the surface expression of CD166 in early stage and cytoplasmic expression in the progressive stage of disease [70]. This may support the contradictory findings of all studies regarding various expressions of CD166 and its clinical significance, described above.

Although the association of CD166 expression with demographic variables of patients was not statistically significant, combined analysis showed the significant association between EpCAM and CD166 expression (*P* = 0.02). Moreover, a significant positive correlation was found between EpCAM/CD166 phenotypes expression and tumor stage (*P* = 0.03), tumor differentiation (*P* = 0.05), neural, and lymph node invasion (*P* =0.01). Thus, co-expression of CD166 with EpCAM (but not alone) was accompanied by a significantly elevated tumor aggressive behavior. Despite a few limitations such as lack of overall survival and follow-up data, our results justify the importance of EpCAM separately and the connection between overexpression of two CSCs markers (EpCAM, CD166) and tumor aggressiveness in CRC tissues. Therefore, management of CRC patients to predict recurrence, relapse, drug resistance, and provide longer survival could come about in the light of using these CSC markers in targeted-therapy strategies.

## Conclusion

Novel molecular therapeutic strategies have shed new light on treatment and found a proper marker on tumorigenic CSCs in the bulk of CRCs and targeting of these cells in order to eradicate them and consequently diminish more of the side effects and damaging processes of non-tumorigenic and normal cells. The results in the current study represented the significant higher expression of EpCAM in tumors with larger size, higher stage, moderate/poor-differentiation, and positive neural, vascular, and lymph node invasion. No correlation was found between CD166 expression and demographic parameters of patients. A link was also seen between EpCAM and CD166 that represented co-expression of two markers (*P* = 0.02) and a significant direct correlation between EpCAM/CD166 phenotypes expression and tumor stage (*P* = 0.03), tumor differentiation (*P* = 0.05), neural, and lymph node invasion (*P* =0.01) in CRC tissues. In other words, CD166, dependently, and EpCAM were identified as putative CSC markers with greater tumor progression and aggressiveness in human CRC specimens.

## Supplementary Information

**Additional file 1 **: **Supplementary Table 1**. Statistical association of EpCAM and CD166 expression with clinicopathological parameters in colorectal cancer logistic analysis. The bolded values are statistically significant.

## Data Availability

The data supporting the conclusion in this study can be obtained by contacting the corresponding authors.

## Abbreviations

CRC: Colorectal cancer
CSCs: Cancer stem cells
EpCAM: Epithelial cell adhesion molecules
ALCAM: Activated leucocyte cell adhesion
TMA: Tissue microarray
IHC: Immunohistochemistry
H&E: Hematoxylin and eosin
H-score: Histochemical score

## References

[1] Bray F, Ferlay J, Soerjomataram I, Siegel RL, Torre LA, Jemal A (2018). Global cancer statistics 2018: GLOBOCAN estimates of incidence and mortality worldwide for 36 cancers in 185 countries. CA Cancer J Clin.

[2] Fan X, Liu L, Shi Y, Guo F, Wang H, Zhao X, Zhong D, Li G (2020). Integrated analysis of RNA-binding proteins in human colorectal cancer. World J Surg Oncol.

[3] Bilchik AJ, DiNome M, Saha S, Turner RR, Wiese D, McCarter M, Hoon DS, Morton DL (2006). Prospective multicenter trial of staging adequacy in colon cancer: preliminary results. Arch Surg.

[4] Manfredi S, Bouvier A, Lepage C, Hatem C, Dancourt V, Faivre J (2006). Incidence and patterns of recurrence after resection for cure of colonic cancer in a well defined population. Br J Surg: Incorp Eur J Surg Swiss Surg.

[5] Li J, Wang Y, Wang X, Yang Q (2020). CDK1 and CDC20 overexpression in patients with colorectal cancer are associated with poor prognosis: evidence from integrated bioinformatics analysis. World J Surg Oncol.

[6] Yang X, Wei W, Tan S, Guo L, Qiao S, Yao B, Wang Z (2021). Identification and verification of HCAR3 and INSL5 as new potential therapeutic targets of colorectal cancer. World J Surg Oncol.

[7] Kadkhoda S, Taslimi R, Noorbakhsh F, Darbeheshti F, Bazzaz JT, Ghafouri-Fard S, Shakoori A (2021). Importance of Circ0009910 in colorectal cancer pathogenesis as a possible regulator of miR-145 and PEAK1. World J Surg Oncol.

[8] Kalantari E, Asadi Lari MH, Roudi R, Korourian A, Madjd Z (2017). Lgr5High/DCLK1High phenotype is more common in early stage and intestinal subtypes of gastric carcinomas. Cancer Biomark.

[9] Rasti A, Mehrazma M, Madjd Z, Abolhasani M, Zanjani LS, Asgari M (2018). Co-expression of cancer stem cell markers OCT4 and NANOG predicts poor prognosis in renal cell carcinomas. Sci Rep.

[10] Shafiei S, Kalantari E, Zanjani LS, Abolhasani M, Lari MHA, Madjd Z (2019). Increased expression of DCLK1, a novel putative CSC maker, is associated with tumor aggressiveness and worse disease-specific survival in patients with bladder carcinomas. Exp Mol Pathol.

[11] Ginestier C, Hur MH, Charafe-Jauffret E, Monville F, Dutcher J, Brown M, Jacquemier J, Viens P, Kleer CG, Liu S (2007). ALDH1 is a marker of normal and malignant human mammary stem cells and a predictor of poor clinical outcome. Cell Stem Cell.

[12] Li C, Heidt DG, Dalerba P, Burant CF, Zhang L, Adsay V, Wicha M, Clarke MF, Simeone DM (2007). Identification of pancreatic cancer stem cells. Cancer Res.

[13] Li T, Su Y, Mei Y, Leng Q, Leng B, Liu Z, Stass SA, Jiang F (2010). ALDH1A1 is a marker for malignant prostate stem cells and predictor of prostate cancer patients’ outcome. Lab Invest.

[14] Wu X-S, Xi H-Q, Chen L (2012). Lgr5 is a potential marker of colorectal carcinoma stem cells that correlates with patient survival. World J Surg Oncol.

[15] Jiang J, Yang P, Guo Z, Yang R, Yang H, Yang F, Li L, Xiang B (2016). Overexpression of microRNA-21 strengthens stem cell-like characteristics in a hepatocellular carcinoma cell line. World J Surg Oncol.

[16] Armstrong A, Eck SL (2003). EpCAM: a new therapeutic target for an old cancer antigen. Cancer Biol Ther.

[17] Shafaei S, Sharbatdaran M, Kamrani G, Khafri S (2013). The association between CD166 detection rate and clinicopathologic parameters of patients with colorectal cancer. Caspian J Intern Med.

[18] Herlyn M, Steplewski Z, Herlyn D, Koprowski H (1979). Colorectal carcinoma-specific antigen: detection by means of monoclonal antibodies. Proc Natl Acad Sci.

[19] Spizzo G, Fong D, Wurm M, Ensinger C, Obrist P, Hofer C, Mazzoleni G, Gastl G, Went P (2011). EpCAM expression in primary tumour tissues and metastases: an immunohistochemical analysis. J Clin Pathol.

[20] Went PT, Lugli A, Meier S, Bundi M, Mirlacher M, Sauter G, Dirnhofer S (2004). Frequent EpCam protein expression in human carcinomas. Hum Pathol.

[21] van der Gun BT, Melchers LJ, Ruiters MH, de Leij LF, McLaughlin PM, Rots MG (2010). EpCAM in carcinogenesis: the good, the bad or the ugly. Carcinogenesis.

[22] Ensinger C, Kremser R, Prommegger R, Spizzo G, Schmid KW (2006). EpCAM overexpression in thyroid carcinomas: a histopathological study of 121 cases. J Immunother.

[23] Went P, Dirnhofer S, Salvisberg T, Amin MB, Lim SD, Diener P-A, Moch H (2005). Expression of epithelial cell adhesion molecule (EpCam) in renal epithelial tumors. Am J Surg Pathol.

[24] Klatte T, Pantuck AJ, Said JW, Seligson DB, Rao NP, LaRochelle JC, Shuch B, Zisman A, Kabbinavar FF, Belldegrun AS (2009). Cytogenetic and molecular tumor profiling for type 1 and type 2 papillary renal cell carcinoma. Clin Cancer Res.

[25] Ralhan R, Cao J, Lim T, MacMillan C, Freeman JL, Walfish PG (2010). EpCAM nuclear localization identifies aggressive thyroid cancer and is a marker for poor prognosis. BMC Cancer.

[26] Seligson DB, Pantuck AJ, Liu X, Huang Y, Horvath S, Bui MH, Han K-R, Correa AJ, Eeva M, Tze S (2004). Epithelial cell adhesion molecule (KSA) expression: pathobiology and its role as an independent predictor of survival in renal cell carcinoma. Clin Cancer Res.

[27] Brunner A, Prelog M, Verdorfer I, Tzankov A, Mikuz G, Ensinger C (2008). EpCAM is predominantly expressed in high grade and advanced stage urothelial carcinoma of the bladder. J Clin Pathol.

[28] Fong D, Steurer M, Obrist P, Barbieri V, Margreiter R, Amberger A, Laimer K, Gastl G, Tzankov A, Spizzo G (2008). Ep-CAM expression in pancreatic and ampullary carcinomas: frequency and prognostic relevance. J Clin Pathol.

[29] Piyathilake CJ, Frost AR, Weiss H, Manne U, Heimburger DC, Grizzle WE (2000). The expression of Ep-CAM (17-1 A) in squamous cell cancers of the lung. Hum Pathol.

[30] Poczatek RB, Myers RB, Manne U, Oelschlager DK, Weiss HL, Bostwick DG, Grizzle WE (1999). Ep-Cam levels in prostatic adenocarcinoma and prostatic intraepithelial neoplasia. J Urol.

[31] Scheunemann P, Stoecklein NH, Rehders A, Bidde M, Metz S, Peiper M, Eisenberger CF (2008). am Esch JS, Knoefel WT, Hosch SB: Occult tumor cells in lymph nodes as a predictor for tumor relapse in pancreatic adenocarcinoma. Langenbecks Arch Surg.

[32] Schmidt M, Hasenclever D, Schaeffer M, Boehm D, Cotarelo C, Steiner E, Lebrecht A, Siggelkow W, Weikel W, Schiffer-Petry I (2008). Prognostic effect of epithelial cell adhesion molecule overexpression in untreated node-negative breast cancer. Clin Cancer Res.

[33] Varga M, Obrist P, Schneeberger S, Mühlmann G, Felgel-Farnholz C, Fong D, Zitt M, Brunhuber T, Schäfer G, Gastl G (2004). Overexpression of epithelial cell adhesion molecule antigen in gallbladder carcinoma is an independent marker for poor survival. Clin Cancer Res.

[34] Went P, Vasei M, Bubendorf L, Terracciano L, Tornillo L, Riede U, Kononen J, Simon R, Sauter G, Baeuerle P (2006). Frequent high-level expression of the immunotherapeutic target Ep-CAM in colon, stomach, prostate and lung cancers. Br J Cancer.

[35] Yang J, Isaji T, Zhang G, Qi F, Duan C, Fukuda T, Gu J (2020). EpCAM associates with integrin and regulates cell adhesion in cancer cells. Biochem Biophys Res Commun.

[36] Zanjani LS, Madjd Z, Axcrona U, Abolhasani M, Rasti A, Asgari M, et al. Cytoplasmic expression of B7-H3 and membranous EpCAM expression are associated with higher grade and survival outcomes in patients with clear cell renal cell carcinoma. Ann Diagn Pathol. 2020:46:151483. 10.1016/j.anndiagpath.2020.151483 32143173

[37] Du W, Ji H, Cao S, Wang L, Bai F, Liu J, Fan D (2013). EpCAM: a potential antimetastatic target for gastric cancer (retraction of vol 55, pg 2165, 2010). Dig Dis Sci.

[38] Scheunemann P, Stoecklein NH, Hermann K, Rehders A, Eisenberger CF, Knoefel WT, Hosch SB (2009). Occult disseminated tumor cells in lymph nodes of patients with gastric carcinoma. A critical appraisal of assessment and relevance. Langenbecks Arch Surg.

[39] Gosens MJ, van Kempen LC, van de Velde CJ, van Krieken JHJ, Nagtegaal ID (2007). Loss of membranous Ep-CAM in budding colorectal carcinoma cells. Mod Pathol.

[40] Ihnen M, Müller V, Wirtz R, Schröder C, Krenkel S, Witzel I, Lisboa B, Jänicke F, Milde-Langosch K (2008). Predictive impact of activated leukocyte cell adhesion molecule (ALCAM/CD166) in breast cancer. Breast Cancer Res Treat.

[41] Mezzanzanica D, Fabbi M, Bagnoli M, Staurengo S, Losa M, Balladore E, Alberti P, Lusa L, Ditto A, Ferrini S (2008). Subcellular localization of activated leukocyte cell adhesion molecule is a molecular predictor of survival in ovarian carcinoma patients. Clin Cancer Res.

[42] Minner S, Kraetzig F, Tachezy M, Kilic E, Graefen M, Wilczak W, Bokemeyer C, Huland H, Sauter G, Schlomm T (2011). Low activated leukocyte cell adhesion molecule expression is associated with advanced tumor stage and early prostate-specific antigen relapse in prostate cancer. Hum Pathol.

[43] Tachezy M, Zander H, Gebauer F, Marx A, Kaifi JT, Izbicki JR, Bockhorn M (2012). Activated leukocyte cell adhesion molecule (CD166)—its prognostic power for colorectal cancer patients. J Surg Res.

[44] Guan S-S, Wu C-T, Liao T-Z, Luo T-Y, Lin K-L, Liu S-H (2020). Indium-111-labeled CD166-targeted peptide as a potential nuclear imaging agent for detecting colorectal cancer stem-like cells in a xenograft mouse model. EJNMMI Res.

[45] Sim SH, Kang M-H, Kim YJ, Lee K-W, Kim D-W, Kang S-B, Eom K-Y, Kim J-S, Lee HS, Kim JH (2014). P21 and CD166 as predictive markers of poor response and outcome after fluorouracil-based chemoradiotherapy for the patients with rectal cancer. BMC Cancer.

[46] Zhang X, Yuan A, Zhao X, Li Z, Cui G (2020). Tumoral expression of CD166 in human esophageal squamous cell carcinoma: implications for cancer progression and prognosis. Cancer Biother Radiopharm.

[47] Horst D, Kriegl L, Engel J, Kirchner T, Jung A (2009). Prognostic significance of the cancer stem cell markers CD133, CD44, and CD166 in colorectal cancer. Cancer Invest.

[48] Lugli A, Iezzi G, Hostettler I, Muraro M, Mele V, Tornillo L, Carafa V, Spagnoli G, Terracciano L, Zlobec I (2010). Prognostic impact of the expression of putative cancer stem cell markers CD133, CD166, CD44s, EpCAM, and ALDH1 in colorectal cancer. Br J Cancer.

[49] Erfani E, Roudi R, Rakhshan A, Sabet MN, Shariftabrizi A, Madjd Z (2016). Comparative expression analysis of putative cancer stem cell markers CD44 and ALDH1A1 in various skin cancer subtypes. Int J Biol Markers.

[50] Kalantari E, Abolhasani M, Roudi R, Farajollahi MM, Farhad S, Madjd Z, Askarian-Amiri S, Mohsenzadegan M (2019). Co-expression of TLR-9 and MMP-13 is associated with the degree of tumour differentiation in prostate cancer. Int J Exp Pathol.

[51] Camp RL, Charette LA, Rimm DL (1943). Validation of tissue microarray technology in breast carcinoma. Lab Invest.

[52] Sadeghi A, Roudi R, Mirzaei A, Zare Mirzaei A, Madjd Z, Abolhasani M (2019). CD44 epithelial isoform inversely associates with invasive characteristics of colorectal cancer. Biomark Med.

[53] Zolfaghari MA, Karimi A, Kalantari E, Korourian A, Ghanadan A, Kamyab K, Esmaili N, Razavi ANE, Madjd Z (2020). A comparative study of long interspersed element-1 protein immunoreactivity in cutaneous malignancies.

[54] Gao F, Zhou B, Xu J-C, Gao X, Li S-X, Zhu G-C, Zhang XG, Yang C (2015). The role of LGR5 and ALDH1A1 in non-small cell lung cancer: cancer progression and prognosis. Biochem Biophys Res Commun.

[55] Ghods R, Ghahremani MH, Darzi M, Mahmoudi AR, Yeganeh O, Bayat AA, Pasalar P, Jeddi-Tehrani M, Zarnani AH (2014). Immunohistochemical characterization of novel murine monoclonal antibodies against human placenta-specific 1. Biotechnol Appl Biochem.

[56] McCarty JK, Miller L, Cox E, Konrath J, McCarty SK (1985). Estrogen receptor analyses. Correlation of biochemical and immunohistochemical methods using monoclonal antireceptor antibodies. Arch Pathol Lab Med.

[57] Roudi R, Korourian A, Shariftabrizi A, Madjd Z (2015). Differential expression of cancer stem cell markers ALDH1 and CD133 in various lung cancer subtypes. Cancer Invest.

[58] Zeki SS, Graham TA, Wright NA (2011). Stem cells and their implications for colorectal cancer. Nat Rev Gastroenterol Hepatol.

[59] Han S, Yang W, Zong S, Li H, Liu S, Li W, Shi Q, Hou F (2017). Clinicopathological, prognostic and predictive value of CD166 expression in colorectal cancer: a meta-analysis. Oncotarget.

[60] Mohtar MA, Syafruddin SE, Nasir SN, Yew LT (2020). Revisiting the roles of pro-metastatic EpCAM in cancer. Biomolecules.

[61] Hiraga T, Ito S, Nakamura H (2016). Ep CAM expression in breast cancer cells is associated with enhanced bone metastasis formation. Int J Cancer.

[62] Meirelles K, Benedict LA, Dombkowski D, Pepin D, Preffer FI, Teixeira J, Tanwar PS, Young RH, MacLaughlin DT, Donahoe PK (2012). Human ovarian cancer stem/progenitor cells are stimulated by doxorubicin but inhibited by Mullerian inhibiting substance. Proc Natl Acad Sci.

[63] Liu D, Sun J, Zhu J, Zhou H, Zhang X, Zhang Y (2014). Expression and clinical significance of colorectal cancer stem cell marker EpCAMhigh/CD44+ in colorectal cancer. Oncol Lett.

[64] Zhou F, Qi Y, Xu H, Wang Q, Gao X, Guo H (2015). Expression of EpCAM and Wnt/β-catenin in human colon cancer. Genet Mol Res.

[65] Goossens-Beumer I, Zeestraten E, Benard A, Christen T, Reimers M, Keijzer R, Sier C, Liefers G, Morreau H, Putter H (2014). Clinical prognostic value of combined analysis of Aldh1, Survivin, and EpCAM expression in colorectal cancer. Br J Cancer.

[66] Huang L, Yang Y, Yang F, Liu S, Zhu Z, Lei Z, Guo J (2018). Functions of EpCAM in physiological processes and diseases (review). Int J Mol Med.

[67] Cherciu I, Bărbălan A, Pirici D, Mărgăritescu C, Săftoiu A (2014). Stem cells, colorectal cancer and cancer stem cell markers correlations. Curr Health Sci J.

[68] Weichert W, Knösel T, Bellach J, Dietel M, Kristiansen G (2004). ALCAM/CD166 is overexpressed in colorectal carcinoma and correlates with shortened patient survival. J Clin Pathol.

[69] Ni C, Zhang Z, Zhu X, Liu Y, Qu D, Wu P, et al. Xu A-x: Prognostic value of CD166 expression in cancers of the digestive system: a systematic review and meta-analysis. PLoS One. 2013;8(8):e70958. 10.1371/journal.pone.0070958 PMC3733726 23940674

[70] Hansen AG, Freeman TJ, Arnold SA, Starchenko A, Jones-Paris CR, Gilger MA, Washington MK, Fan K-H, Shyr Y, Beauchamp RD (2013). Elevated ALCAM shedding in colorectal cancer correlates with poor patient outcome. Cancer Res.

