# Supplementary material for: Significant co-expression of putative cancer stem cell markers, EpCAM and CD166, correlates with tumor stage and invasive behavior in colorectal cancer
Source: World J Surg Oncol. 2022 Jan 11;20:15. doi: 10.1186/s12957-021-02469-y (PMC8751119; doi:10.1186/s12957-021-02469-y)
Supplement: Supplementary file 1 — Additional file 1 : Supplementary Table 1. Statistical association of EpCAM and CD166 expression with clinicopathological parameters in colorectal cancer logistic analysis. The bolded values are statistically significant. [file 12957_2021_2469_MOESM1_ESM.docx]

Statistical association of EpCAM and CD166 expression with clinicopathological parameters in colorectal cancer logistic analysis. The bolded values are statistically significant.

| Variables | Total No(%) | EpCAM expression  (Mean H-score = 196) | | *P-value* | CD166 expression    (Mean H-score = 83) | | *P-value* |
| --- | --- | --- | --- | --- | --- | --- | --- |
|  |  | OR (95% CI) | |  | OR (95% CI) | |  |
| **Mean age years** |  |  |  |  |  |  |  |
| 60≥ | 241(52.5) | 0.625 (0.392- 0.998) | | **0.049** | 1.034 (0.660- 1.618) | | 0.885 |
| 60< | 218(47.5) |  |  |  |  |  |  |
| **Gender** |  |  |  |  |  |  |  |
| Male | 236(51.5) | 1.378 (0.865- 2.196) | | 0.177 | 0.753 (0.481- 1.179) | | 0.215 |
| Female | 222(48.5) |  |  |  |  |  |  |
| **Tumor size (cm)** |  |  |  |  |  |  |  |
| 5≥ | 300(66) | 0.699 (0.427- 1.143) | | 0.154 | 1.110 (0.685- 1.798) | | 0.672 |
| 5< | 154(34) |  |  |  |  |  |  |
| **TNM stage** |  |  |  |  |  |  |  |
| I | 71(16) | 31.969 (1.219- 838.674) | | **0.038** | 0.571 (0.059- 5.506) | | 0.628 |
| IIA | 172(38) |  |  |  |  |  |  |
| IIB | 21(5) |  |  |  |  |  |  |
| IIIA | 76(17) |  |  |  |  |  |  |
| IIIB | 68(15) |  |  |  |  |  |  |
| IIIC | 17(4) |  |  |  |  |  |  |
| IVA | 21(5) |  |  |  |  |  |  |
| **Tumor location** |  |  |  |  |  |  |  |
| Cecum | 70(16.5) | 0.963 (0.823- 1.126) | | 0.634 | 1.052 (0.906- 1.222) | | 0.503 |
| Sigmoid | 140(34) |  |  |  |  |  |  |
| Rectom | 114(27) |  |  |  |  |  |  |
| Colon ascending | 34(8) |  |  |  |  |  |  |
| Colon transvers | 29(7) |  |  |  |  |  |  |
| Colon descending | 12(3) |  |  |  |  |  |  |
| Rectosigmoid | 18(4.5) |  |  |  |  |  |  |
| **Tumour differentiation** |  |  |  |  |  |  |  |
| Well | 165(36.5) | 0.540 (0.331- 0.881) | | **0.014** | 1.324 (0.834- 2.102) | | 0.234 |
| Moderate/Poor | 289(63.5) |  |  |  |  |  |  |
| **Distant metastasis** |  |  |  |  |  |  |  |
| Positive | 25(6) | 0.168 (0.015- 1.946) | | 0.154 | 1.486 (0.279- 7.905) | | 0.643 |
| Negative | 416(94) |  |  |  |  |  |  |
| **Neural Invasion** |  |  |  |  |  |  |  |
| Positive | 90(20) | 1.508 (0.785- 2.897) | | 0.217 | 1.221 (0.616- 2.421) | | 0.568 |
| Negative | 355(80) |  |  |  |  |  |  |
| **Vascular invasion** |  |  |  |  |  |  |  |
| Positive | 69(15.5) | 1.296 (0.577- 2.909) | | 0.530 | 0.970 (0.447- 2.107) | | 0.939 |
| Negative | 379(84.5) |  |  |  |  |  |  |
| **Lymph node invasion** |  |  |  |  |  |  |  |
| Positive | 171(37.5) | 1.064 (0.455- 2.489) | | 0.887 | 0.715 (0.302- 1.694) | | 0.446 |
| Negative | 286(62.5) |  |  |  |  |  |  |
